# Mechanistic and therapeutic insight into the effects of cinnamon in polycystic ovary syndrome: a systematic review

**DOI:** 10.1186/s13048-021-00870-5

**Published:** 2021-10-09

**Authors:** Vahid Maleki, Amir Hossein Faghfouri, Fatemeh Pourteymour Fard Tabrizi, Jalal Moludi, Sevda Saleh-Ghadimi, Hamed Jafari-Vayghan, Shaimaa A. Qaisar

**Affiliations:** 1 Clinical Cancer Research Center, Milad General Hospital, Tehran, Iran; 2 Knee and Sport Medicine Research Center, Milad Hospital, Tehran, Iran; 3 Student Research Committee,Tabriz University of Medical Sciences, Tabriz, Iran; 4 Nutrition Research Center, Faculty of Nutrition and Food Sciences, Tabriz University of Medical Sciences, Tabriz, Iran; 5 Faculty of Nutritional Sciences and Food Technology, Kermanshah University of Medical Sciences, Kermanshah, Iran; 6 Department of Nutrition, Faculty of Health, Arak University of Medical Sciences, Arak, Iran; 7 Chemistry Department, College of Education, University of Garmian, Sulimmania, Iraq

**Keywords:** Cinnamon, Polycystic Ovary Syndrome, Lipid Profile, Insulin Resistance, Oxidative Stress

## Abstract

Polycystic ovary syndrome (PCOS) is one of the most common endocrine diseases in the women at their reproductive age. Nowadays, the use of herbal compounds for lesser side effects, as compared to drug treatments, has become popular for the prevention and reduction of the complications of this disease. Evidence suggests that cinnamon, given its antioxidant and anti-inflammatory properties, can be associated with reduced metabolic complications from chronic non-communicable diseases. This systematic review aimed to determine the potential effect of cinnamon on the metabolic status in the PCOS. PICO framework for current systematic review was Population (P): subjects with PCOS; Intervention (I): oral cinnamon supplement; Comparison (C): the group as control or administered placebo; and Outcome (O): changed inflammatory, oxidative stress, lipid profile, glycemic, hormonal and anthropometric parameters and ovarian function. PubMed, Scopus, EMBASE, ProQuest and Google Scholar were searched from their very inception until January, 2020, considering specific keywords to explore the related studies. Out of 266 studies retrieved by the search strategy, only nine were eligible for evaluation. All clinical trials, animal studies, and published English-language journal studies were eligible for this review. The results showed that increased high-density lipoprotein and insulin sensitivity were increased by the cinnamon supplementation while low-density lipoprotein, triglyceride, and blood glucose were decreased in patients with PCOS. However, the results related to the potential effects of cinnamon on body weight and body mass index were inconsistent, thus calling for further studies. Also, despite improved results regarding the effect of cinnamon on oxidative stress and ovarian function, further studies are required to explore the precise mechanisms. Overall, the effects of cinnamon on the improvement of metabolic status in PCOS were promising. However, to observe clinical changes following cinnamon supplementation in PCOS, more clinical trials with higher doses of cinnamon and a longer duration of intervention are needed.

## Introduction

Polycystic ovary syndrome (PCOS) is the most common endocrine disorder in the women at the reproductive age and before their menopause; prevalence is estimated to be between 5 and 20% in the world [1]. PCOS is characterized by clinical signs and symptoms such as irregular menstruation (oligomenorrhea, dysmenorrhea and amenorrhea), hirsutism, severe acne, androgenic alopecia, hyperandrogenism and infertility [2]. The pathophysiology of PCOS is not precisely defined because it is a complex and multiple etiology affected by a set of genetic and environmental factors [3]. In general, the main roles of PCOS pathogenesis are hyperandrogenism and hyperinsulinemia, both stimulating each other [4, 5]; this leads to several metabolic abnormalities including obesity, insulin resistance, dyslipidemia, type 2 diabetes, dyslipidemia, increased inflammation and oxidative stress [6, 7].

Nowadays, the use of herbal compounds, and complementary and alternative therapies to prevent, treat and reduce the complications of PCOS or to moderate the use of drugs and their side effects has become widespread [8, 9]. Cinnamon is one of the popular healthful herbal compounds worldwide; its medical properties have been extensively explored [8, 10, 11]. Cinnamon may act as a promising agent in the treatment of PCOS by increasing the activity of phosphatidylinositol 3-kinase in the insulin signaling pathway, thus potentiating insulin action [12]. In line with this, increasing evidence shows that the presence of inositol is necessary for insulin sensitivity and activity, which could be regarded as a crucial effect in the patients with PCOS. They mediate insulin-stimulated glucose utilization in the cell by different pathways including the cellular uptake of glucose or glycogen synthesis; additionally, several components of the metabolic syndrome, including glucose tolerance, blood pressure and triglyceride levels, are improved by inositol. Additionally, inositol may have a role in restoring fertility in women with PCOS. Myo-inositol as an inositol is a second messenger of the luteinizing hormone (LH) and follicle-stimulating hormone (FSH) signaling pathways in oocytes and follicular cells. Follicular maturity and oocyte quality are determined by the concentrations of myo-inositol in human follicular fluid [13, 14].

Cinnamon is a *Cinnamomum*bark 5 to 7 m tall; it is always green too [15]. Cinnamon has several components (procyanidins, diterpenes, phenylpropanoids, mucilage and polysaccharides), but the most important part is cinnamaldehyde [16]. Studies have shown that cinnamon has anti-oxidant [17], anti-inflammatory [18], anti-diabetic [19, 20], antifungal and antibacterial properties [21], as well as improving nausea and diarrhea [22]. It is one of the most popular spices consumed by people all over the world and also there is a wide over-use of this type of herbal medicine in PCOS. Despite several studies that have already evaluated the potential effects of cinnamon on weight changes, glycemic control, dyslipidemia, ovarian hormones, androgen levels, biomarkers of inflammation, oxidative stress and other metabolic variables in PCOS, there is no comprehensive systematic review summarizing the outcomes of these previous studies. So, the aim of this study was to explore the knowledge gaps addressed and recommendations provided for the future research regarding the impact of cinnamon on PCOS.

## Methods

### Search strategy

This study was designed based on Preferred Reporting Items for Systematic Reviews and Meta-Analyses (PRISMA) protocol for reporting systematic reviews and meta-analyses. A literature search was conducted to find related studies in PubMed, SCOPUS, Embase, ProQuest, and Google Scholar databases using keywords, ProQuest and Google Scholar electronic databases using the keywords “Cinnamon”[Title/Abstract] OR “Cinnamons” [Title/Abstract] OR “Camellia sinensis” [Title/Abstract] OR “Cinnamomum verum” [Title/Abstract] OR Cinnamomum [Title/Abstract] OR “Cinnamomum zeylanicum” [Title/Abstract] OR “Cinnamomum” [Title/Abstract] **AND** “polycystic ovary syndrome”[Title/Abstract] OR “PCOS” [Title/Abstract] OR “sclerocystic ovary syndrome” [Title/Abstract] OR “dysmetabolic syndrome”[Title/Abstract]. Reference lists and related records were manually reviewed. There are no restrictions on the timing and type of studies in the search strategy. Only articles published in English journals until January 2020 were reviewed. PICO framework for current systematic review was Population (P): subjects with PCOS; Intervention (I): oral cinnamon supplement; Comparison (C): the group as control or administered placebo; and Outcome (O): changed inflammatory, oxidative stress, lipid profile, glycemic, hormonal and anthropometric parameters and ovarian function.

### Eligibility criteria

The eligibility criteria for entering the study were as follows: (1) all clinical trials, (2) animal studies, and (3) (1) in vitro models (2) published in English-language journals; studies with insufficient information were excluded.

### Data extraction

At first, after receiving full text studies eligible for this study, they were screened by two investigators independently. Data extraction was done using a standardized data collection form and research questions. In the next step, the following information was extracted from each eligible study: first author’s name, year of publication, country of origin, type of study, methods or models, quantity and gender of participants, dosage and duration of the intervention applied, and the main outcomes. If there were a disagreement between the researchers, the accuracy and quality of the included data would be evaluated by a third party.

## Results

### Selected articles

**Fig. 1:**
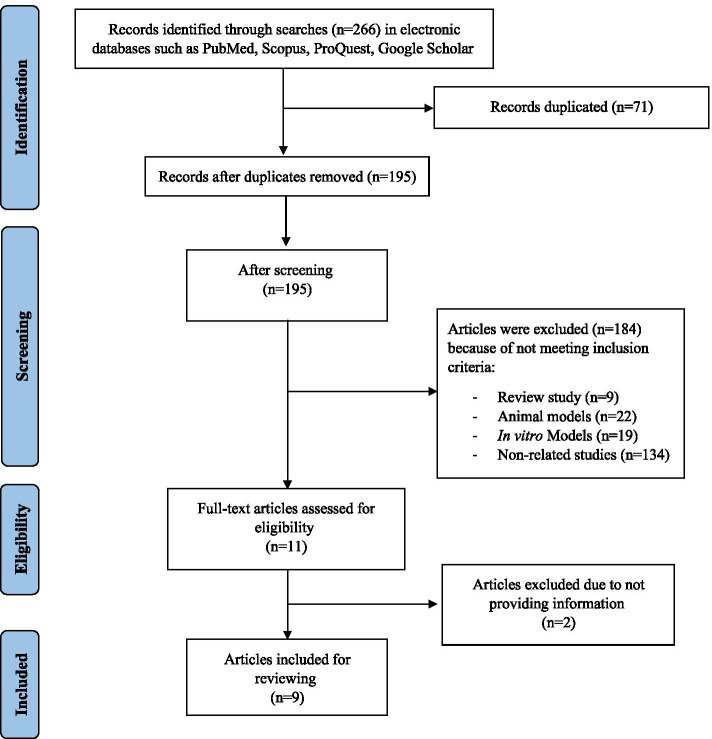
Flow diagram of the literature search and study selection process

Flowchart of the process applied for selecting the studies is summarized in Fig. 1. Regarding the potential effects of cinnamon on the metabolic parameters in PCOS, we found 266 publications by initially using the search strategy; based on this, 71 of them were duplicates, resulting in 195 non-duplicated publications. Of these, 184 articles were excluded. After that, 2 articles were excluded due to insufficient information (*i.e.*, letters, comments, short communication, conferences, congresses and abstracts). Finally, nine papers were selected for inclusion in this systematic review Table 1.

**Table 1:** Characteristics of studies that reported the effects of cinnamon on PCOS

Type of study	Authors/date	Model	Intervention (Daily dose)	Duration	Outcome
**Animal**	Dou et al./ 2018 [23]	60 Prepubertal C57BL/6 mice (PCOS mice model induced by DHEA; age 25 days) Were randomly divided into three groups (control group, DHEA group, DHEA + cinnamon group)	Cinnamon powder (10 mg/100 g body weight mixed in 100 μL 0.5% methyl cellulose) via gavage	20 days	Cinnamonin PCOS mice model: Restores the estrous cyclicity and ovary morphology Improves insulin sensitivity and reduce insulin resistance Mitigate impaired glucose tolerance Down-regulate and reduce testosterone and LH levels Reduce IGF-I level and increase IGFBP-1 level in plasma as well as in ovary tissue
	Khodaeifar et al./ 2019 [24]	64 female Wistar rats weighing 200–250 g (PCOS was induced by a single dose injected estradiol valerate) divided into 8 groups (three type treatment: A. graveolens (200 mg/kg) extract Cinnamon (200 mg/kg) extract Cinnamon (200 mg/kg) + A. graveolens (200 mg/ kg) extracts	Cinnamon extract 200 mg/kg orally	14 days	Treatment with *Cinnamon. zeylanicum* extracts was found to: Decrease the level of FBS and Insulin Decline the insulin resistance Reduce serum level of LDL, TG, and cholesterol and an increase HDL level Had antioxidant properties and reduce the oxidative stress and protect the ovarian tissue from the oxidative damage
**Human**	Borzoei et al./ 2017 [25]	Double-blind RCT 84 overweight or obese PCOS patients (20–38 years)	Cinnamon powder 1.5 g /day (3 cinnamon capsules; each one contained 500 mg)	8 weeks	Cinnamon significantly: increased serum TAC (↑9.28%) Decreased MDA (↓7.87%) Improved serum level of lipids: increased HDL levels (5.49%) and decreased total cholesterol (**↓**7.73%) and LDL (**↓**10.24%) and non-significant changes in serum TG (**↓**18.24%) Non-significant differences between groups in BMI
	Borzoei et al./ 2018 [10]	Double-blind RCT 84 overweight or obese PCOS patients (20–38 years)	Cinnamon powder 1.5 g /day (3 cinnamon capsules; each one contained 500 mg)	8 weeks	Cinnamon significantly: Decreased serum fasting blood glucose (**↓**10.63%), insulin (**↓**12.63%), HOMA-IR (**↓**20.25%), total cholesterol and LDL and weight Increased HDL (compared with placebo) Decreased Serum TG and BMI (in comparison with baseline values), but non-significant between two groups Non-significant changes in serum adiponectin
	Wang et al./ 2007 [26]	Pilot RCT study, fifteen women with PCOS (with mean BMI 28.8 ± 1.3 kg/m^2^ and mean age 31.1 ± 2.0 years)	Daily oral 1 g cinnamon extract (1 capsule containing 333 mg of cinnamon extract / 3 times per day)	8 weeks	In cinnamon group: BMI, total T, and E2 remained the same and unchanged Fasting glucose decreased (-16.9%), QUICKI increased significantly (7.7%), HOMA-IR decreased (-44.5%) and improved insulin sensitivity
	Wiweko et al. / 2017 [27]	Double-blind RCT 38 PCOS participants were divided into two groups: metformin (20 patients) and DLBS3233(18 patients)	DLBS3233 (herbal extracts combination of *Lagerstroemia speciosa* and *Cinnamomum burmannii* 100 mg / day	6 months	In the DLBS3233 group: A significant decrease in the serum AMH level (changes were lower in compared to metformin group) A significant decrease of BMI (This effect was not observed in the metformin group)
	Salehpouret al / 2015 [28]	Double-blind RCT 112 obese and PCOS adolescent girls (12.6–17 years old)	Cinnamon extract (500 mg twice daily) or metformin (500 mg twice daily)	1 year	Cinnamon does not differ from metformin in decreasing the insulin resistance and Apo B: Apo A1 ratio, but decrease the BMI less
	Kort et al. / 2014 [29]	Double-blind RCT 45 PCOS women (aged 18–38 years)	1500 mg/day cinnamon capsules	6 months	In the cinnamon group: -Significant improvement in menstrual cyclicity (from baseline and also, compared to controls) -Non-significant change of serum androgen, SHBG levels, and measures of insulin resistance in either group (cinnamon and placebo) over the study period -Non-significant change of weight, subcutaneous fat thickness, and ovarian volume in either group (cinnamon and placebo) over the study period
	Hajimonfarednejad et al./ 2017 [30]	Double-blind RCT 66 PCOS women (aged 18–45 y; BMI ≥ 18)	Cinnamon powder capsules 1.5 g/day in 3 divided doses	12 weeks	Daily cinnamon supplementation resulted in: **-**Non-significant reduction of all anthropometric factors (weight, BMI, and waist circumference), FBS, 2-h postprandial blood glucose, TG, Chol, and serum androgen levels **-**Significant reduction of fasting insulin, HOMA-IR, LDL, and HDL (in comparison with the placebo group)

*Abbreviations*: *AMH* anti-Mullerian hormone, *Apo A1* apolipoprotein A1, *Apo B* apolipoprotein B, *BMI* body mass index, *DHEA* dehydroepiandrosterone, *E2* Estradiol, *FBS* Fasting blood sugar, *HDL* high-density lipoprotein, *HOMA-IR*, homeostatic model assessment for insulin resistance, *IGF-1* Insulin-like growth factor 1, *IGFBP-1* Insulin-like growth factor-binding protein, *IR* insulin resistance, *LDL* low-density lipoprotein, *LH* luteinizing hormone, *MDA* Malondialdehyde, *PCOS* Polycystic Ovary Syndrome, *QUICKI* quantitative insulin sensitivity check index, *RCT* Randomized controlled trial, *SHBG* sex hormone binding globulin: Testosterone, *TAC* total antioxidant capacity, *TC* total cholesterol, *TG* triglyceride

### Risk of bias

The risk of bias within the randomized control trials (RCT) was evaluated using the Cochrane scale by two independent reviewers (FPF and VM); the scores are represented in Table 2. This tool comprises seven selection bias items (random sequence generation and allocation concealment), performance bias, detection bias, attrition bias, reporting bias, and other forms of bias. Any discrepancies were resolved upon consultation with a third reviewer (HJV). We investigated the risk of bias for seven included studies that covered the human subject. All studies were of the low risk of bias.

**Table 2:** Assessment of the risk of bias

Auhtors	Borzoei et al. 2017 [25]	Borzoei et al. 2018 [10]	Wang et al. 2007 [26]	Wiweko and Susanto 2017 [27]	Salehpour et al. 2015 [28]	Kort et al. 2014 [29]	Hajimonfared nejad et al. 2017 [30]
Random sequence generation (selection bias)	**L**	**L**	**L**	**L**	**L**	**L**	**L**
Allocation concealment (selection bias)	**L**	**L**	**L**	**L**	**L**	**L**	**L**
Blinding of participants and personnel (performance bias)	**L**	**L**	**L**	**L**	**L**	**L**	**L**
Blinding of outcome assessment (detection bias)	**L**	**M**	**U**	**L**	**L**	**U**	**U**
Incomplete outcome data (attrition bias)	**L**	**L**	**L**	**U**	**L**	**U**	**U**
Selective reporting (reporting bias)	**M**	**L**	**U**	**L**	**U**	**L**	**L**
Other bias	**L**	**L**	**L**	**U**	**U**	**L**	**U**
Overall quality	**L**	**L**	**L**	**L**	**L**	**L**	**L**

*L* low risk of bias, *M* Moderate risk of bias, *U* Unknown

### Biological activities of Cinnamon

Cinnamon, as a multifaceted medicinal plant, has been shown to have antihyperlipidemic [31], hepatoprotective [32], anti-obesity [33], anti-diabetic [34], anti-oxidative [33], and anti-inflammatory properties [18]. The potential glucose-lowering effect and its pharmacological mechanisms as insulin potentiating factors have been recognized in several previous studies [35, 36]. It has been found that cinnamic acid improves glucose tolerance in vivo and stimulates insulin secretion in vitro [36]. In addition, the polyphenolic compounds of cinnamon-like kaempferol, rutin, quercetin and catechin display insulin-like properties [37, 38]. Procyanidin polyphenol type-A polymers extracted from cinnamon stimulate the autophosphorylation of the insulin receptor and inhibit protein tyrosine phosphatase I. Autophosphorylation of the insulin receptor kinase and the subsequent phosphorylation of its principal substrate were found to be decreased or inhibited in the insulin-responsive tissues of obese or non-insulin-dependent diabetes subjects [39, 40]. Accordingly, cinnamon mitigates insulin resistance and enhances glucose utilization by increasing phosphatidylinositol 3-kinase activity in the insulin signaling pathway, thus potentiating the insulin action [12]. The anti-obesity activity of cinnamon has been confirmed by the observed effects of cinnamaldehyde, another active component of cinnamon, on the pre-adipocyte differentiation. It was found that lipid accumulation was reduced, and the expression of peroxisome proliferator-activated receptor-γ (PPAR-γ) was down-regulated significantly by cinnamaldehyde. In addition, it could up-regulate AMP-activated protein kinase (AMPK) and acetyl-CoA carboxylase [41, 42]. Cinnamon is rich in antioxidants because of its high levels of different phytochemicals compounds with free radical scavenger actions and antioxidant activities, such as proanthocyanidins, epicatechin, gamma-terpinene, phenol, camphene, salicylic acid, eugenol and tannins. These compounds decrease oxidative stress by the inhibition of 5-lipoxygenase [43]. Hence, the anti-oxidative nature and components of cinnamon determine its anti-oxidative activities [11, 44].

### Cinnamon and weight changes in PCOS

In comparison to their non-PCOS counterparts, PCOS patients were more likely to suffer from obesity; this exacerbates many aspects of the PCOS related phenotype, especially cardiovascular risk factors such as glucose intolerance and dyslipidemia [45]. Several studies have investigated the probable effects of cinnamon intake on anthropometric indices (including weight, body mass index (BMI) and waist circumference of the PCOS sufferers. In one animal study, cinnamon powder intake (10 mg/100 g body weight) for 20 days had no significant effect on body weight; further, no differences were shown in the body weight of mice among the three groups [23]. Also, in a pilot RCT, the daily consumption of 1 g of the cinnamon extract (CE) for eight weeks did not affect the BMI of the PCOS women significantly [26]. On the contrary, in a double-blind RCT, a significant decrease of BMI was observed following the intake of the herbal extracts combination containing cinnamon (100 mg/daily) for six months [27]. Another study also reported that there was no significant difference regarding BMI between the metformin consumer group and the cinnamon consumer one; however, the treatment with 1000 mg CE for 12 months significantly decreased BMI, as compared to placebo [28]. In the study done by Hajimonfared Nejad et al. during the 12-week treatment period, supplementation with 1.5 g daily cinnamon resulted in a reduction of all anthropometric factors (weight, BMI and waist circumference); however, these changes were not statistically significant [30]. Similarly, no significant changes in the weight and subcutaneous fat thickness were observed in one other study after consumption of 1500 mg/day cinnamon capsules for 6 months [29]. In addition, an Iranian team [10, 25] found that BMI was decreased significantly in the cinnamon group, in comparison with the baseline values; however, decreases in BMI in the two groups were not significant after the intervention. Despite this, a significant decrease was observed in the body weight of the subjects by cinnamon supplementation. They reported that weight loss in the studied subjects was not sufficient to decrease BMI, as compared to placebo.

### Cinnamon and glycemic control in PCOS

Due to the insulin resistance in the pathophysiology of PCOS and the established utility of insulin-sensitizing agents in the treatment of PCOS, as well as the available evidence showing that cinnamon can reduce insulin resistance and treat insulin-resistant diabetes, cinnamon has been proposed as a possible alternative therapy for the PCOS patients [30]. Therefore, several studies have investigated the probable effects of the cinnamon intake on the glycemic indices of PCOS sufferers. Plasma levels of fast blood sugar (FBS) and insulin were significantly higher in the PCOS group, as compared to the control. In an animal study done by Khodaeifar et al., the CE intake (200 mg/kg) for 14 days by the PCOS rats resulted in significant reductions of the plasma levels of FBS and insulin, as compared to the placebo group [24]. Glucose tolerance test and insulin tolerance test state were investigated following cinnamon intake in an animal study done by Dou et al. They reported that impaired glucose tolerance in PCOS mice was likely to be mitigated by cinnamon treatment (10 mg/100 g body) for 20 days [23]. The results of an RCT conducted by Borzoei et al. [10] also showed a significant reduction of serum fasting blood glucose, insulin and homeostatic model assessment for insulin resistance (HOMA-IR), as compared to the placebo group, by consuming cinnamon powder (1.5 g /day) for eight weeks in the PCOS patients. However, no significant changes were seen in serum adiponectin in either group. So, their results indicated improved serum glycemic indices and no change of serum adiponectin due to the cinnamon supplementation of the PCOS women [10]. Similarly, the results of other RCTs showed that cinnamon supplementation of the PCOS women resulted in a significant reduction of fasting glucose, insulin and insulin resistance, as measured by various indices and improvement of insulin sensitivity [26, 28, 30]. Salehpour et al. concluded that cinnamon did not differ from metformin in decreasing the insulin resistance [28]. However, some studies [29, 30] have reported that reduction in FBS, 2-h postprandial blood glucose, and measures of insulin resistance was not statistically significant.

### Cinnamon and dyslipidemia in PCOS

Dyslipidemia is the most common metabolic abnormality in PCOS, as 70% of the PCOS patients exhibit abnormal serum lipid [46]. One animal study [24] and four RCTs [10, 25, 28, 30] investigated the effect of cinnamon intake on the lipid profile in the PCOS subjects. With some differences in the reported results, they all indicate the improvement effect of cinnamon on the lipid profiles. Khodaeifar et al. [24] also reported the significant reducing effect of cinnamon intake on the plasma levels of cholesterol, low-density lipoprotein (LDL) and triglyceride (TG). Also, the decreased level of high-density lipoprotein (HDL), which is common in the PCOS subjects, was enhanced significantly due to cinnamon intake, as compared to that of the control group. However, an Iranian team [10, 25] found that cinnamon supplementation (1.5 g /day) improved the serum lipid profile in the women with PCOS; despite this, the changes in the serum TG were not significant. In addition, Salehpour et al. [28] reported that the consumption of the CE (1000 mg /day) for 12 months resulted in a significant reduction in the apolipoprotein B: apolipoprotein A1 ratio. In the study done by Hajimonfared Nejad et al. [30], during the 12-week treatment period, supplementation with 1.5 g daily cinnamon resulted in a non-significant reduction of serum TG and chol, while the decrease of LDL was significant. So, they reported that cinnamon supplementation (1.5 g/day) for 12 weeks improved insulin sensitivity and decreased insulin and LDL level in the women with PCOS.

### Cinnamon, hormones and ovarian function in PCOS

Hyperinsulinemia arising from insulin resistance is associated with a higher capacity of ovarian androgen production [46]. Excessive ovarian androgen production contributes to the pathogenesis of PCOS. On the other hand, insulin resistance underlies the hallmark symptoms of PCOS, such as androgen excess and menstrual irregularity [47, 48]. Due to the improvement effect of cinnamon on the insulin sensitivity, it seems that it could be advantageous in the modification of ovarian hormones and androgens by mitigating insulin resistance. The effects of cinnamon treatment on the reproductive features, including ovarian hormones, gonadotropins, estrous cycle, androgens, and ovarian morphology and histology, have been investigated in the previous studies [23, 24, 26, 27, 29, 30]. Other than an animal study [23], others [26, 29, 30] have reported that cinnamon intake by the PCOS women have no significant effect on the blood levels of androgens (testosterone, dehydroepiandrosterone sulfate), SHBG and Estradiol (E2). Kort et al. [29] also investigated the effect of cinnamon on menstrual cyclicity in the women with PCOS, reporting that cinnamon intake (1500 mg/day) for six months improved menstrual cyclicity significantly (from the baseline in the cinnamon group, as also compared to the controls). In these PCOS women, by measuring ovulatory progesterone levels in the luteal phase, ovulatory menses were confirmed. Dou et al. [23] also reported that cinnamon had the ability to down-regulate the serum levels of LH and testosterone, to restore the estrous cyclicity, and to recover the ovary morphology induced by the PCOS state (recovery rate 68%). It was also shown that the ovarian tissue of the PCOS subjects was damaged due to the production of cystic follicles and atretic body in the ovary; also, a decline in the number of the normal follicles was observed. All of these were owing to hyperandrogenism [49]. In this regard, Khodaeifar et al. [24] also reported the protective effect of the CE on the ovarian tissue damages induced by the PCOS. This treatment led to the reduction of the number of atretic follicles, while enhancing the normal follicles. Another hormone involved in the pathogenesis and insulin resistance in the pathophysiology of PCOS is the Anti-mullerian hormone (AMH). Serum AMH level was found to be higher in the PCOS cases, as compared to the controls [50]. With a high AMH level, folliculogenesis may be suppressed because follicle sensitivity to FSH is decreased [51]. In the study carried out by Wiweko and Susanto [27], the herbal extract combination of the CE (100 mg/day) caused a significant decrease in the serum AMH level, but this change was lower when compared to the metformin group. In addition, Insulin-like growth factor 1(IGF-1) had a negative impact on normal folliculogenesis and ovulation [52, 53]. Accordingly, one animal study [23] demonstrated that cinnamon powder (10 mg/100 g body) for 20 days decreased the IGF-1 level, while it increased the Insulin-like growth factor-binding protein (IGFBP-1) level in plasma, as well as in the ovary, according to the PCOS mice model.

### Cinnamon and oxidative stress in PCOS

Metabolic and endocrine disturbances such as hyperinsulinemia, hyperandrogenism and dyslipidemia might be responsible for developing PCOS-associated oxidative stress. So, PCOS is proposed as a significantly decreased antioxidant status, and the resultant oxidative stress induces an inflammatory environment [42, 54]. Khodaeifaret al. [24] also demonstrated that treatment with the *Cinnamon. Zeylanicum* extract (200 mg/kg) had antioxidant properties, reducing oxidative stress and protecting the ovarian tissue from oxidative damage in the PCOS model rats. In an RCT developed by Borzoei et al. [25], the 1.5 g /day cinnamon powder intake for eight weeks significantly increased the serum total antioxidant capacity (increased 9.28%), while it decreased malondialdehyde (by 7.87%), as compared to the placebo. It was also shown that cinnamon supplementation had antioxidant potency, improving oxidative stress in the PCOS women. These findings were all in agreement with the previous study, which reported that the treatment with the cinnamon. zeylanicum extract, due to its antioxidant property, could protect the ovarian tissue from the oxidative damage [24].

## Discussion

PCOS, as one of the most common endocrine disorders, has many metabolic complications. These include dyslipidemia, insulin resistance, obesity and overweight, and hormonal abnormalities [55]. PCOS is also related to elevated oxidative stress levels in the body [56]. Various compounds can affect the metabolic status and oxidative conditions of this disease, including cinnamon or compounds found in CEs such as cinnamic acid and cinnamaldehyde [23].

Insulin resistance is one of the major factors involved in the pathophysiology of PCOS [57]. Several studies have investigated the effect of cinnamon on glycemic indices and insulin sensitivity in the PCOS patients. In most studies, cinnamon has been shown to have an ameliorative effect on blood glucose and insulin sensitivity [10, 23, 24, 26, 28]. In two studies, despite insignificant results, cinnamon was shown to reduce FBS, 2-h postprandial blood glucose and measures of insulin resistance [29, 30]. In the study done by Kort et al., these results might be related to the high sample loss during the follow-up period (more than 50%) [29]. In the study conducted by Hajimonfarednejad et al., cinnamon powder reduced HOMA-IR significantly; however, the results pertaining to FBS 2-h postprandial blood glucose were insignificant [30]. The insignificant results on glycemic indices may be related to the lower levels of these indices before supplementation among the included subjects. So, cinnamon supplementation could not be much effective on these indices.

**Fig. 2:**
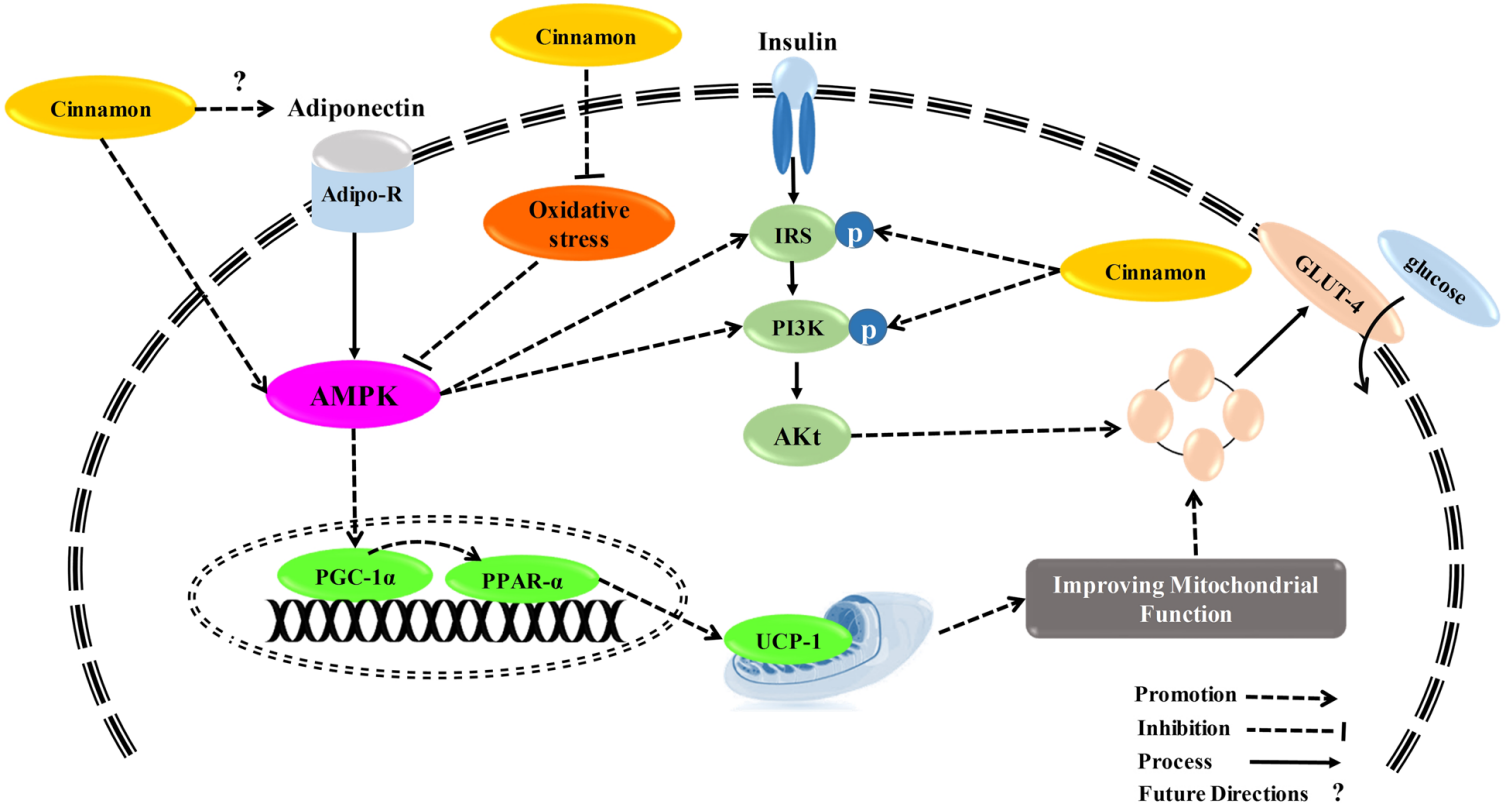
Possible mechanisms of the cinnamon on insulin signaling. Abbreviations: IRS: Insulin Receptor Substrate, PI3K: Phosphoinositide 3-kinase, AKt: Protein kinase B, PGC-1α: Peroxisome proliferator activated receptor gamma coactivator 1-alpha, PPAR-α: Peroxisome proliferator-activated receptor alpha, AMPK: AMP-activated protein kinase

CE improves insulin resistance through the enhancement of insulin action. CE can increase the insulin receptor (IR) β, and IR substrate-1 (IRS1) tyrosine phosphorylation and IRS1/phospho-inositide 3-kinase (PI3K) contents in the skeletal muscle of rats after 3-week treatment [58]. Another in vitro study done by Nikzamir et al. showed that the cinnamaldehyde treatment of mouse muscle cells led to an increase in glucose transporter-4 (GLUT4) gene expression, resulting in the elevation of glucose uptake [59]. In streptozotocin-induced diabetic rats, CE treatment led to the enhancement of uncoupling protein-1(UCP-1) and GLUT4 content in their brown adipose tissues and muscles [60]. A suggested way for the translocation of GLUT4 by cinnamon is the activation of the AMP-activated protein kinase (AMPK) [61], as shown in Fig. 2. In one pathway, CE or other conditions like energy depletion can stimulate the liver kinase B1 (LKB-1) enzyme; LKB-1 directly activates/phosphorylates AMPK [62, 63]. Kopp et al. also revealed that trans-cinnamic acid could act as a ligand for the G-protein-coupled receptor, thereby stimulating AMPK signaling [64]. Besides GLUT4 translocation, activated AMPK has other effects on carbohydrate metabolism, like inhibition of gluconeogenesis through suppressing key enzymes, namely, glucose-6-phosphatase and phosphoenolpyruvate-carboxy-kinase [65]. In a recent study, Aras et al. suggested that cinnamon could decrease blood glucose by increasing in the nerve growth factor (NGF) levels [66]. NGF increase is related to an increase in insulin level and regulation of pancreatic β cell homeostasis [66]. Forkhead box protein O1 (FOXO1) as a transcription factor has been implemented in lipid localization and GLUT4 expression and activity. It has been recently revealed that FOXO1 is a potential target of CE [67].

However, the results related to the effects of cinnamon on the anthropometric indices in the PCOS patients have been inconsistent. While some in vivo and RCT studies have not observed a significant improvement effect [23, 25, 26, 29], others have found that cinnamon could ameliorate the anthropometric measures in the PCOS patients [10, 27, 28]. Despite this, the positive effect of cinnamon on the anthropometric measures cannot be ignored. Two RCT studies on metabolic syndrome and diabetic patients have confirmed that cinnamon has a beneficial effect on weight, BMI and waist circumference [68, 69]. These contradictory results in different studies on the PCOS patients could be due to the difficulty of adjusting the various variables affecting weight. Also, the basic characteristics of the study participants could be effective in the final results. As well, for being effective in anthropometric measures, the high dosage of CE or cinnamon in a long duration must be administered. As a result, further studies with better inclusion and exclusion criteria are needed to reach more accurate conclusions.

Obesity is associated with an increased risk of developing insulin resistance in the PCOS patients. Therefore, factors that may have a beneficial effect on one can affect the other one as well [70, 71]. For instance, in an in vivo study done on diabetic rats, cinnamon treatment could improve body weight and fat mass, as well as increasing the insulin level [72]. As discussed above, cinnamon can stimulate AMPK. Canto et al. also showed that activated AMPK could regulate the expression of the genes involved in energy expenditure in the skeletal muscle, resulting in the activation of the catabolic pathways that produce ATP [73]. It was proposed that CE decreased TG in overweight women through the activation of PPAR-γ, as a key regulator of adipogenesis, in turn affecting adiponectin and very low-density lipoprotein metabolism [74].

Interestingly, cinnamon can be effective in food consumption and gastrointestinal processes. Transient receptor potential ankyrin 1 or TRPA1 is an ion channel protein expressed in the sensory nerves. Recently, is has been shown that TRPA1 is expressed on the epithelial cells of the gastrointestinal tract [75]. Tamura et al. also revealed that cinnamaldehyde could act as a TRPA1 agonist, resulting in the decrease of the visceral fat of the mice fed with high fat and high sucrose diet [76]. Stimulated TRPA1 regulates gastrointestinal motility, gastric emptying, and cholecystokinin release through serotonergic pathways [77, 78]. Also, the activated TRPA1 increases the UCP-1 expression in the brown adipose tissue and induces autonomic thermoregulation [76, 79]. Moreover, TRPA1 and ghrelin are co-localized in the enteroendocrine cells of the duodenum. Therefore, activation of TRPA1 by cinnamaldehyde can lead to the lower secretion of ghrelin (hunger hormone), resulting in the lower food consumption [80].

Except for some minor differences, all studies have, therefore, highlighted the beneficial effect of cinnamon on the lipid profile [10, 24, 25, 28, 30]. The same results were driven from studies on metabolic syndrome patients, but not diabetic patients [68, 69]. Cinnamon increases the expression of PPARgamma/alpha, as well as the target genes related to lipid homeostasis such as lipoprotein lipase (LPL) and CD36 in 3T3-L1 adipocyte, resulting in the improved lipid profile [42]. Moreover, the activated AMPK suppresses acetyl coA-carboxylase and induces malonyl-coA-dehydrogenase in lipid metabolism, resulting in an increase of beta-oxidation and a decrease of fatty acid biosynthesis [65]. Recently, stimulatory effect of CE on adipocyte browning has been demonstrated. CE increased the gene expression of markers related to brown adipocyte including Cidea, Prdm16, Pgc, and Cpt-1; while decreased that of white adipocyte markers including Dpt and Igf in 3T3-L1 adipocytes [81].

Androgen production is elevated in the PCOS patients. Insulin resistance is a common cause of elevated androgen production in these patients [47, 48]. Therefore, cinnamon, through the improvement of insulin resistance, may help to regulate androgen production. Also, excessive advanced glycation end products (AGEs) in the PCOS patients may lead to higher androgen products; cinnamon, via decreasing these products, down-regulates androgen products in the PCOS patients [82, 83]. However, the results obtained in regard to the cinnamon effect on androgen production in different studies have not been the same. For example, while an in vivo study supports the beneficial effect of cinnamon on the hormonal regulation in the PCOS model [23], three RCT studies did not come up with any promising results [26, 29, 30]. BMI, weight and fat mass might affect the way androgen production responds to cinnamon supplementation. Because the majority of the PCOS patients have obesity, patients may need a high dosage of CE in a long duration to overcome insulin resistance and therefore, excessive androgen production. Khodaeifar et al. have also demonstrated that cinnamon protects the ovarian tissue from oxidative damage, thus regulating the ovarian function in PCOS [24]. This study, thus, revealed new insights into the effect of cinnamon on PCOS, showing its antioxidant effect.

PCOS is an oxidative condition. Elevated amounts of reactive oxygen species (ROS) are found in PCOS; these are related to its pathogenesis [84]. Cinnamic acid has four side chains (R group) in its structure. Depending on the structure of these chains, cinnamic acid has antioxidant, antibacterial, antiviral and antifungal properties [85]. Also, cinnamaldehyde derivatives, because of having reductive groups like hydroxyl and methyl, have radical scavenging properties [86]. One RCT study showed that Cinnamon. Zeylanicum extract supplementation could protect the ovarian tissue from oxidative damage [24]. Another RCT also demonstrated that cinnamon could enhance the antioxidant capacity in the PCOS patients [25], which was like another study on diabetic patients [87]. The effects of cinnamon on sirtuin-1 (SIRT1), with a wide range of functions including regulation of immune system and inflammation, have been suggested recently [87]. Wang et al. revealed that cinnamtannin D1 (CD1) exhibited an antiapoptosis activity in palmitic acid-treated pancreatic β cells via alleviating oxidative stress in vitro. This protective effect of CD1 against glucolipotoxicity in pancreatic β cells was mediated by enhancement of autophagy in vivo and in vitro through AMPK/mTOR/ULK1 pathway [88].

Our study is the first comprehensive systematic review focusing on the effect of cinnamon on PCOS. However, our study had some limitations. First, due to few studies done on each of the studied factors, conclusive findings about the beneficial effects of cinnamon on PCOS should be taken with precaution. Moreover, a limited range of cinnamon dosages has been administered in included studies. Therefore, we could not reach a conclusion about the safety of different dosages of cinnamon. As a result, more studies with different designs are needed in the future. Second, we included only published English-language studies and were not able to evaluate articles published in other languages.

### Knowledge gaps and future directions

Studies considering the effects of cinnamon and CE on PCOS have partly pointed out the positive effects of cinnamon on some metabolic processes and oxidative stress in these patients. Therefore, cinnamon, especially in higher dosages (1.5 g/day), can be considered as a new therapeutic agent in the treatment of PCOS. Clinical implications of cinnamon have been more impressive on the lipid profile and glycemic parameters. However, the results have been inconsistent in some cases. As many factors are related to PCOS, future RCT studies need to include a large number of participants, considering more precise inclusion and exclusion criteria, to reach more accurate results in a specific population. While the effect of the CE on some mediators which activate AMPK has been investigated in different conditions, the study of these pathways in the PCOS models seems to be necessary. Also, the role of other mediators like SIRT1 in activating AMPK by CE in the PCOS patients can be investigated in the future studies. Caglayan et al. also showed that the SIRT1 level was higher in the PCOS patients [89].

Moreover, the role of TRPA1 as a factor related to many metabolic processes is not completely known in the PCOS related complications, calling for additional studies. Modulation of some mediators’ production like PPARs and AGEs products with cinnamon in the PCOS models can be considered in the future studies. Resveratrol, another polyphenolic compound, decreases androgen production through the regulation of critical enzymes in androgen production, like CYP17 and CYP21, or other signaling pathways such as Akt/PKB [90, 91]. Future studies can also consider the cinnamon effect on these factors to describe the clear mechanism of androgen production regulation by cinnamon.

Another factor contributing to some inconsistent results in clinical researches is related to the heterogeneity of the disease. In fact, women with PCOS are not equally affected. Therefore, in designing clinical studies on PCOS, it is proposed to consider the heterogeneity and different phenotypes of the syndrome, in addition to various clinical manifestations which may lead to different results (the concept of splitter vs lumpers) [92]. Given that the glucose metabolism is an important aspect of PCOS, further research should emphasize on different phenotypes and also study the role of insulin resistance in prognosis, diagnosis and treatment.

## Conclusion

According to the results of this systematic review, cinnamon supplementation improved lipid profile by significant reduction of LDL and TG level and elevation of HDL. Also, cinnamon supplementation improved glucose homeostasis by increasing the insulin sensitivity. However, due to impact of several factors on weight and BMI, the results related to the effects of cinnamon on the anthropometric indices in patients with PCOS were inconsistent; so, further studies are needed. Furthermore, despite promising results obtained in regard to the effects of cinnamon on oxidative stress and ovarian function, these effects have not yet been well addressed.

## Data Availability

Not applicable.
